# Detecting and responding to deterioration of a baby during labour: surveys of maternity professionals to inform co-design and implementation of a new standardised approach

**DOI:** 10.1136/bmjopen-2024-084578

**Published:** 2025-03-06

**Authors:** Jan W van der Scheer, Margaret Blott, Mary Dixon-Woods, Annabelle Olsson, Jordan Moxey, Sarah Kelly, Matthew Woodward, Giulia Maistrello, Wendy Randall, Sarah Blackwell, Chloe Hughes, Caroline Walker, Louise Dewick, Rachna Bahl, Tim J Draycott, Thiscovery Authorship Group, André Sartori, ABC Contributor Group, Bethan Everson, Jenni Burt

**Affiliations:** 1 THIS Institute (The Healthcare Improvement Studies Institute), University of Cambridge, Cambridge, UK; 2 Royal College of Obstetricians and Gynaecologists, London, UK; 3 RAND Europe, Cambridge, UK; 4 The Royal College of Midwives, London, UK; 5 North Bristol NHS Trust, Westbury on Trym, UK; 6 University Hospitals Bristol and Weston NHS Foundation Trust, Bristol, UK

**Keywords:** Health & safety, NEONATOLOGY, OBSTETRICS, Quality Improvement, Surveys and Questionnaires, Capacity Building

## Abstract

**Abstract:**

**Objectives:**

Detecting and responding to deterioration of a baby during labour is likely to benefit from a standardised approach supported by principles of track-and-trigger systems. To inform co-design of a standardised approach and associated implementation strategies, we sought the views of UK-based maternity professionals.

**Design:**

Two successive cross-sectional surveys were hosted on an online collaboration platform (Thiscovery) between July 2021 and April 2022.

**Setting:**

UK.

**Participants:**

Across both surveys, 765 UK-based maternity professionals.

**Primary and secondary outcome measures:**

Count and percentage of participants selecting closed-ended response options, and categorisation and counting of free-text responses.

**Results:**

More than 90% of participants supported the principle of a standardised approach that systematically considers a range of intrapartum risk factors alongside fetal heart rate features. Over 80% of participants agreed on the importance of a proposed set of evidence-based risk factors underpinning such an approach, but many (over 75%) also indicated a need to clarify the clinical definitions of the proposed risk factors. A need for clarity was also suggested by participants’ widely varying views on thresholds for actions of the proposed risk factors, particularly for meconium-stained liquor, rise in baseline fetal heart rate and changes in fetal heart rate variability. Most participants (>75%) considered a range of resources to support good practice as very useful when implementing the approach, such as when and how to escalate in different situations (82%), how to create a supportive culture (79%) and effective communication and decision-making with those in labour and their partners (75%).

**Conclusions:**

We found strong professional support for the principle of a standardised approach to detection and response to intrapartum fetal deterioration and high agreement on the clinical importance of a set of evidence-based risk factors. Further work is needed to address: (1) clarity of clinical definitions of some risk factors, (2) building evidence and agreement on thresholds for action and (3) deimplementation strategies for existing local practices.

**Strengths and limitations of this study**

- The online surveys gathered insights from maternity professionals to guide the initial stages of co-designing better clinical practices, helping to support successful future implementation.
- The use of an online collaboration platform enabled wide and user-friendly participation of maternity professionals.
- The wide participation of various professionals across the UK—recruited through a range of stakeholder networks—is a strength, though not every UK maternity unit was represented.
- The sample is limited to the UK only—views of professionals in other countries might be different.

## Introduction

 Failure to consistently detect and respond to deterioration of a baby during labour is a remarkably persistent problem in the UK^1^,^3^ and in health systems worldwide.^4^,^6^ Lack of accurate and timely recognition of babies at risk of developing brain injury during labour can have devastating consequences for families and result in major litigation claims.^26^,^9^ One challenge is that methods of assessing risk of intrapartum fetal deterioration tend to focus primarily on changes in the fetal heart rate,^10^ as identified using either intermittent auscultation or cardiotocography (CTG).^1 2^ Not only is there wide variation in practices relating to fetal heart rate monitoring during labour,^2 11 12^ excess focus on fetal heart rate features risks overlooking the relevance of other developing risk factors (eg, delay in labour, meconium-stained liquor, vaginal bleeding, maternal fever and tachysystole).^10 13 14^

Consistent detection and response to intrapartum fetal deterioration is likely to benefit from a standardised approach that systematically considers a set of evidence-based risk factors alongside fetal heart rate patterns.^1015^,^17^ Use in practice of such an approach could be strengthened by evidence-based application and implementation of principles of track-and-trigger systems similar to those used in other clinical areas.^18^,^21^ Such systems are based on the principle that there may be periods during which clinical deterioration is detectable by ‘tracking’ a predefined set of clinical risk factors over time, with specific thresholds ‘triggering’ action.^22^ Also critical, in a maternity setting, is engagement with the person in labour and their birth partner to identify concerns that may indicate risks, assure their agency in making decisions and demonstrate respect.^323^,^25^

Based on these insights, the Avoiding Brain Injury in Childbirth (ABC) programme was commissioned by the Department of Health and Social Care in 2021, with the aim of developing and standardising an evidence-based, co-designed approach to detection and response to intrapartum fetal deterioration in England.^26^,^28^ Standardisation helps avert unwarranted variation and clinical risks that may otherwise arise when varying approaches are used within and between healthcare units.^29^,^35^ However, standardisation is not straightforward to achieve,^29^,^3335^ as illustrated by challenges in attempts to standardise track-and-trigger systems across national health systems.^3738 40^,^43^ Success in standardisation is more likely when developers work collaboratively with those intended to use a new system, rather than imposing it.^2631 43^,^53^ One way to undertake standardisation collaboratively is through the application of co-design principles,^26 54^ including early and continued engagement of healthcare professionals during development of a new system.^26 31 45 49 51 53^ Such engagement may generate new perspectives sensitising development and implementation and enhance feelings of ownership of professionals intended to use the system in practice.^26 45 54^ We sought the views of UK maternity professionals to:

1. Assess support for a proposed standardised approach that systematically considers a range of developing intrapartum risk factors alongside fetal heart rate features to help detect fetal deterioration.
2. Identify perceptions of the importance and clarity of a proposed set of evidence-based risk factors to underpin a standardised approach.
3. Elicit views on thresholds for action for responding to suspected intrapartum fetal deterioration.
4. Explore ways to support the implementation of a standardised approach.

## Methods

We conducted two successive cross-sectional surveys of maternity professionals working in the UK. The surveys were hosted on Thiscovery, a secure online collaboration platform (https://www.thiscovery.org/about).

### Questionnaires

We developed two questionnaires in consultation with a multidisciplinary group of maternity professionals and survey experts and pilot-tested them prior to launch. Both questionnaires used a combination of closed-ended and free-text questions.

The first questionnaire (see online supplemental file 1) aimed to assess support for a standardised approach that systematically considers a range of developing intrapartum risk factors alongside fetal heart rate features to help detect fetal deterioration. The questionnaire also asked about the importance and clarity of definitions of a proposed set of evidence-based risk factors^10 13 14^ and elicited suggestions for implementation of a standardised approach.

Findings of the first questionnaire informed the design of the second (see online supplemental file 2). This questionnaire aimed to explore professionals’ views on definitions and thresholds for action for responding to suspected deterioration of a baby during labour.^10 13 14^ The questionnaire also asked views of participants on national and international guidelines for fetal heart rate monitoring^11^ and assessed the perceived usefulness of resources to support good practice when implementing a standardised approach.^55^

### Participants

Qualified healthcare professionals providing maternity care or working in policy, research or other contexts relevant to maternity within the UK were eligible to take part in both surveys. Participants in the first survey were recruited via multiple routes, including advertising through relevant professional organisations and networks, and targeted emails to eligible registered users of Thiscovery. The same methods were used to recruit participants for the second survey, supplemented by recontacting consented first survey participants. There was no minimum or maximum specified sample size.

### Data collection

Survey 1 was open from July to November 2021 and survey 2 from February to April 2022. The recommended standards for fetal monitoring in the UK during this period were the National Institute for Health and Care Excellence (NICE) guidelines from 2014 (updated 2017),^56^ prior to recent updates.^10 16 17^ Before taking part, participants read an information sheet, confirmed their eligibility and provided online written consent.

### Data analysis

Analyses were conducted separately for each survey, confined to data of participants who had completed all closed-ended questions. For questions with closed-ended response options, we undertook descriptive analysis supported by Excel and R statistical software. For free-text responses, our analysis was based on principles of content analysis,^57^ using a four-step process. First, the multidisciplinary authorial team generated categories based on the topics and questions of the survey. Second, two health services research analysts coded all free-text entries to one of the categories. If a participant’s free-text entry included more than one suggestion or comment, the entry was split and coded as separate responses. Third, two analysts generated subcategories ‘bottom-up’ (ie, data-driven) based on participant responses. Clinical authors verified clinical accuracy of use of the subcategories. Finally, one analyst counted the number of responses and the number of participants providing those responses within categories and subcategories. Another analyst verified these counts.

### Patient and public involvement

Although there was no direct patient and public involvement in these surveys, maternity service users were engaged and involved in the development of a proposed approach to detection and response to intrapartum fetal deterioration.^25 26^

## Results

A total of 765 maternity professionals took part across both surveys—553 participants completed survey 1 and 342 participants completed survey 2, with 130 participants completing both surveys (table 1).

**Table 1:** Characteristics of participants who completed survey 1 (n=553) and/or survey 2 (n=342)

	Survey one	Survey two
	n (%)	n (%)
Professional role		
Midwife Band 5–7	286 (52)	217 (64)
Midwife Band 8–9	47 (9)	13 (4)
Trainee/registrar/trust doctor obstetricians	46 (8)	24 (7)
Consultant obstetrician	112 (20)	74 (22)
Other	62 (11)	14 (14)
Clinical leadership role		
Yes	169 (31)	162 (47)
No	383 (69)	179 (52)
Did not report	1 (0)	1 (0)
Type of maternity unit/setting*		
Obstetric unit	475 (66)	321 (94)
Alongside midwifery unit	114 (16)	82 (24)
Freestanding midwifery unit	21 (3)	11 (3)
Community	63 (9)	34 (10)
Other	48 (7)	12 (4)
Did not report	1 (0)	0 (0)
Region of work		
East of England	48 (9)	23 (7)
London	58 (11)	38 (11)
Midlands	86 (16)	56 (16)
North East and Yorkshire	72 (13)	46 (14)
North West	72 (13)	57 (17)
Northern Ireland	14 (3)	7 (2)
Scotland	12 (2)	7 (2)
South East	55 (10)	53 (16)
South West	82 (15)	37 (11)
Wales	30 (5)	12 (4)
Did not report	24 (4)	6 (2)

* Multiple response options possible. Alongside: A midwifery-led unit or birth centre situated in the same hospital or on the same site as an obstetric unit. Free-standing: A midwifery-led unit or birth centre not situated in a hospital or site with an obstetric unit.

### Survey 1

#### Support for a standardised approach

Most participants (95%, n=543) supported or strongly supported a systematic approach consisting of monitoring not only fetal heart rate features but also other developing risk factors during labour. A similarly large proportion (94%; n=520) agreed or strongly agreed that there was a need for a standardised approach to be used across all maternity units and settings in the UK.

#### Suggestions for development and implementation of a standardised approach

Free-text responses drew attention to the challenges associated with current lack of standardisation of some practices within and between maternity units, and the potential benefits of reducing variations in practice and quality of care. Synthesis of 165 free-text responses yielded the following suggestions for development and implementation of a standardised approach:

- Highlight the evidence base informing the approach.
- Showcase the extent to which it improves clinical outcomes.
- Include the use of clinical judgement.
- Ensure transferability across different maternity settings.
- Make the approach user-friendly and practical.
- Facilitate local adaptations where needed.
- Consider the views of multiple stakeholders (including maternity professionals and service users).

#### Perceived importance of proposed risk factors

**Figure 1:**
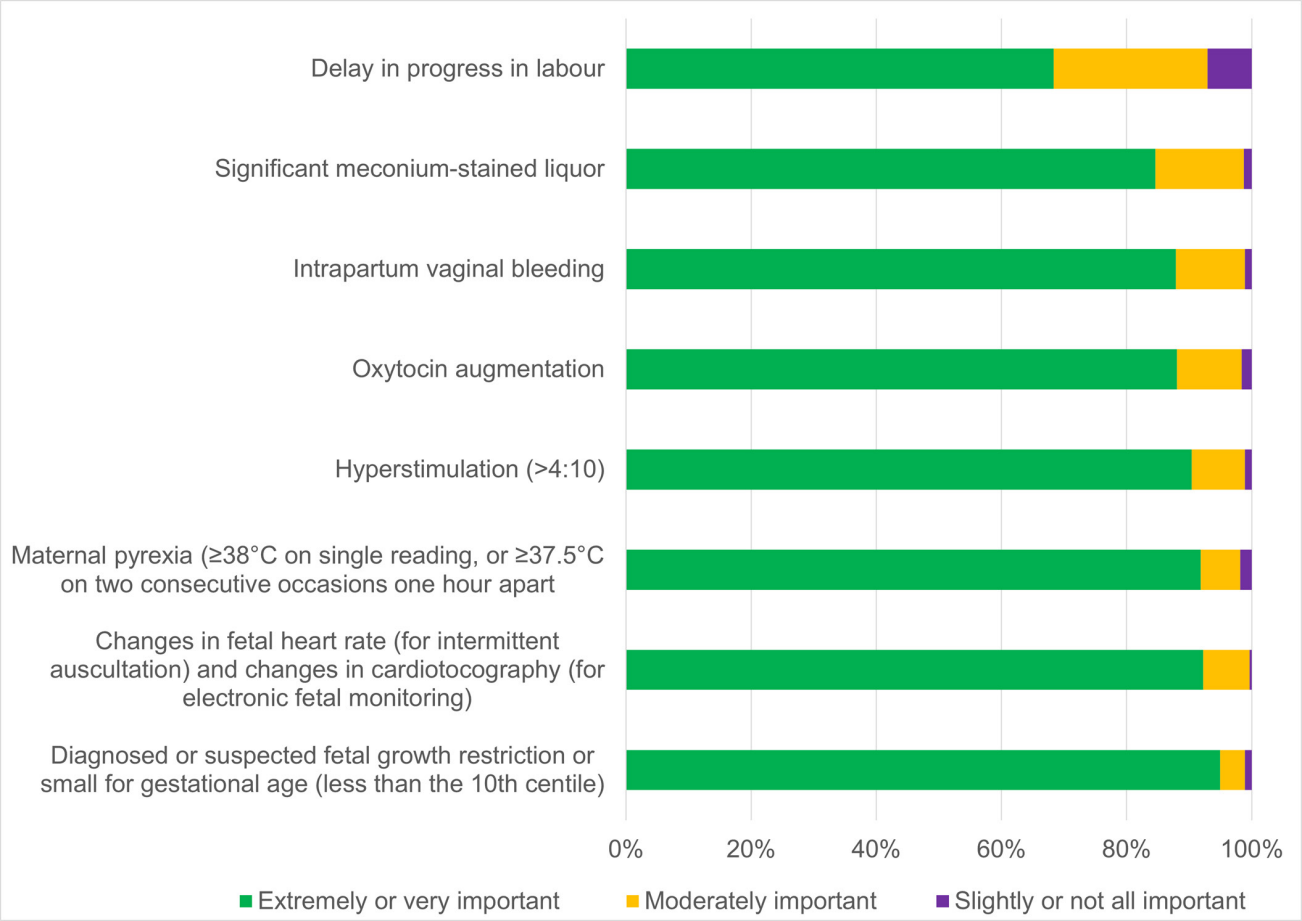
Participants’ (n=553) perceived importance of proposed risk factors for detecting and responding to suspected fetal deterioration during labour.

Most participants (over 80%) indicated that seven of the eight proposed risk factors for deterioration of a baby during labour were extremely or very important (figure 1). The eighth risk factor (delay in progress of labour) was considered extremely or very important by 69% of participants, with a further 25% seeing it as moderately important.

#### Perceived clarity of clinical definitions of risk factors

Over 75% of the participants (n=415) identified at least one of the proposed risk factors as requiring improvement in terms of their clinical definitions, such as in the definitions of fetal heart rate changes (75%, n=415), delay in progress in labour (63%, n=347), meconium-stained liquor (60%, n=331) and vaginal bleeding (52%, n=286). Synthesis of 295 free-text responses identified a range of suggestions for improving the definitions, including:

- Significant meconium-stained liquor (ie, consider amending from ‘significant’ meconium to ‘presence’ of meconium).
- Intrapartum vaginal bleeding (ie, distinguish between vaginal bleeding and a ‘show’).
- Delay in progress of labour (ie, specify what is meant by ‘delay’ and consider the wider context in which the delay occurs).

### Survey 2

#### Meconium

Participants varied in their preferences for how to describe meconium representing a ‘low risk’ or ‘high risk’ to the baby. For example, some referred to low-risk meconium as ‘thin’ (32%, n=108) while others used ‘insignificant’ (24%, n=82), while high-risk meconium was referred to by some as ‘thick’ (28%, n=108) or ‘significant’ (33%, n=111). About one-third of the participants did not think there was a need to differentiate between types of meconium. Over half of the participants (58%, n=197) agreed that the presence of ‘any’ meconium found in a low-risk setting using intermittent auscultation should prompt advising or offering immediate transfer to obstetric-led care. Approximately 40% indicated that a transfer should be dependent on the presence of other risk factors, such as fetal heart rate concerns or maternal fever. Free-text responses indicated difficulties in differentiating between the appearance of meconium and associated risks.

#### Vaginal bleeding

Most participants (91%, n=312) indicated that ‘fresh vaginal bleeding’ should be classified as intrapartum bleeding requiring action to be taken. A majority (79%, n=270) also indicated that ‘heavily soaked pads’ should trigger action.

#### Delay in progress of labour

Most participants (91%, n=311) reported using definitions for delay in labour offered in national guidelines from NICE available at the time of the survey (see online supplemental file 2).^56^ Only 39% (n=133) considered these definitions appropriate. A further 35% (n=119) indicated that the NICE definitions needed improvement, while 26% (n=90) were unsure. Synthesis of 117 free-text responses suggested that participants perceived the definitions to focus too much on cervical dilatation without sufficiently considering other factors such as contractions, rotation or descent.

Dilatation appears to be the main focus with delay, however a change in fetal position descent etc, may actually outweigh the perceived slow progress in dilatation. As always its multifactorial. (Midwife)

#### Maternal pyrexia

Approximately two-thirds of the participants (68%, n=234) indicated that maternal fever (ie, ≥38°C on a single reading or ≥37.5°C on two consecutive readings 1 hour apart) should trigger escalation of care. Other participants’ responses indicated that the trigger for action should refer to 2 hours apart (14%, n=47).

#### Tachysystole

Most participants (85%, n=291) indicated that five or more contractions in 10 min require action to be taken. Over 90% of the participants indicated that use of oxytocin (98%, n=334), resting tone (95%, n=326) and length of each contraction (91%, n=310) should also be considered.

#### Rise in fetal heart rate baseline

Participants’ views varied on how to express a significant rise in the baseline above the initial baseline rate at the start of labour. They most frequently selected 20 beats per minute (19%, n=65), 10% rise (39%, n=133), 15% rise (12%, n=41) or 20% rise (11%, n=38). Free-text responses highlighted professionals’ difficulties in defining a simple and meaningful threshold.

Difficult to assign a number, as a small increase may be just as significant, with a gradual rise. (Midwife)

#### Reduced variability on a CTG trace

Almost half of the participants (46%, n=156) defined a significant period of reduced variability on a CTG trace as 50 to 90 min, while 35% of participants defined it as 30–50 min (table 2). The presence of other risk factors, such as meconium or sepsis, shortened the period of reduced variability deemed to trigger action. Free-text responses related to the assessment of the significance of a period of reduced variability in light of other risk factors.

**Table 2:** Participants’ (n=342) views on what constitutes a significant period of reduced variability in cardiotocography results

	Significant period	Significant period in the presence of other risk factors
	n (%)	n (%)
Any period of reduced variability	3 (1)	51 (15)
Less than 30 min	3 (1)	32 (9)
30–50 min	119 (35)	173 (51)
50–90 min	156 (46)	46 (14)
More than 90 min	18 (5)	2 (1)
Other	43 (13)	38 (1)

Difficult to quote a time frame for reduced variability; need to look at other features (Consultant obstetrician)

#### Increased variability on a CTG trace

Views varied widely on the time period of increased variability on a CTG trace that should prompt further action. Responses ran across the full spectrum of options provided, ranging from any period of increased variability (13%, n=44), to 11–15 min (15%, n=52), to a period of more than 30 min (17%, n=59). Free-text responses (n=30) suggested that the significance of increased variability was seen as dependent on presence of other risk factors and the stage of labour.

#### Decelerations of the fetal heart rate

In a situation of hearing a deceleration during intermittent auscultation, no participant indicated that they would continue with usual labour care. About a fifth would transfer to obstetric-led care (22%, n=74). Over two-thirds (69%, n=237) would increase the frequency of auscultation, listening immediately following the next three contractions to assess for further decelerations. Considering CTG trace changes as risk factors, most participants indicated that prolonged decelerations/bradycardia should be defined by a period of more than 3 min (90%, n=308). Of the types of decelerations that might cause concern and prompt re-examination of the full clinical picture, majorities of participants selected the following: fetal heart rate spends more time decelerating when compared with the baseline rate (98%, n=335), decelerations with a slow return to baseline after a contraction (U-shaped) (97%, n=332), late deceleration(s) (93%, n=319) and decelerations lasting 60 s or more (89%, n=305).

#### Guidelines for fetal heart rate monitoring

Table 3 shows that fewer than half of the participants (46%) reported using national NICE guidelines, that is, those available at the time of the survey,^56^ in their current place of work. Less than one-fifth (18%) indicated they would choose to follow these guidelines by preference, while over one-third (35%) preferred ‘physiological’ approaches (table 3). The 41 free-text responses referred to local practices that combine various guidelines, concerns about the variation and lack of standardisation in use of guidelines across various units and fear of litigation creating feelings of uncertainty.

**Table 3:** Participants’ (n=342) use of, and preferences, for guidelines for electronic fetal monitoring

	Used in current place of work	Preferred
	n (%)	n (%)
NICE	156 (46)	60 (18)
FIGO	29 (9)	20 (6)
Physiological	47 (14)	118 (35)
NICE and FIGO	20 (6)	17 (5)
NICE and Physiological	47 (14)	70 (21)
FIGO and Physiological	26 (8)	39 (11)
Other	17 (5)	18 (5)

The survey was conducted before recent NICE guidelines updates.^10 16 17^

FIGO, International Federation of Gynaecology and ObstetricsNICE, National Institute for Health and Care Excellence

It is really difficult to ascertain what is the best thing to do is, when clinical research on each of the areas is so limiting. The fear of litigation is high […]. (Midwife)

#### Good practice resources

Over three-quarters of the participants identified one or more of the following topics as very or extremely useful to address in resources for good practice to support detection and response to fetal deterioration:

- When and how to escalate in different situations (n=280, 82%).
- How to create a supportive culture (n=271, 79%).
- Effective teamwork to support detection and response to possible fetal deterioration (n=266, 78%).
- Situational awareness (n=263, 77%).
- How to deal with disagreements (n=260, 76%).
- Effective communication and decision-making with those in labour and their partners (n=256, 75%).

Many also considered that it would be extremely or very useful to have resources on features of safe maternity units (n=206, 64%), and how to gain professional confidence in new tools supporting the standardised approach (n=228, 66%).

## Discussion

### Main findings

In these two surveys of 765 UK-based maternity professionals, we found strong support for the principle of a standardised approach to detect and respond to intrapartum fetal deterioration, and high agreement on the clinical importance of a proposed set of evidence-based risk factors. These findings indicate good potential for successful implementation of a standardised approach in a clinical area with wide variation in practices and where people may feel a sense of local ownership about their own approach. The surveys also highlighted the following issues to address in further co-design of the standardised approach and associated implementation strategies:

1. A need to improve the clarity of clinical definitions of some of the proposed risk factors.
2. Widely varying views on thresholds for actions of the proposed risk factors, particularly for meconium-stained liquor, rise in baseline fetal heart rate and changes in fetal heart rate variability.
3. Highly variable reported use of, and attitudes towards, national and international guidelines for electronic fetal monitoring.

### Need for undertaking standardisation collaboratively

Failure to harmonise safety procedures at the system level may create the conditions for adverse outcomes in maternity care.^3 29 58 59^ Although complex to achieve,^29^,^3336^ standardisation to systematically encode best practice is a promising strategy for reducing risk of harm.^34 35^ Consistent with experience in other track-and-trigger systems,^20^ standardisation has potential to reduce unwarranted variation and improve practice,^35 39 47 48^ in particular when adopting a suitable collaboration-based approach.^26 31 45 60^

Lack of consensus in the field^11 12^ likely contributes to the widely varying views on thresholds for action regarding risk factors and the various attitudes towards national and international guidelines. Similar challenges also underpinned the work of others developing clinical tools to better integrate fetal heart rate features and other risk factors when assessing fetal status during labour.^61 62^ This variation in perspectives also highlights the potential tensions between standardisation and a preference for locally established approaches^29^—such as those that people are most familiar with or consider most appropriate.^12^ Previous research suggests that suboptimal adoption of standardised track-and-trigger systems is often driven by insufficient consideration of acceptability and usability of healthcare professionals during development and implementation.^29 37 44 46 63 64^ Addressing these factors is therefore a critical aspect of co-designing any new approach^26^ and was a key motivation for our study.

Although tensions about clinical best practices are often not easily resolved,^29 32 33^ they are likely to benefit from engaging and involving maternity professionals throughout the lifecycle of developing new approaches.^26 31 45 49 51 53^ Involvement of front-line staff may also help address the challenges posed by the sociotechnical complexity of detecting and responding to intrapartum fetal deterioration,^12^ especially when the involvement would include agreement around adaptative strategies when services are under pressure.^65^,^67^ Our findings illustrate that early consultation can help assess the level of agreement on proposed changes, identify areas of uncertainty, ambivalence and concern, and incorporate users’ perspectives and priorities into design.^26 49^ Further co-design that addresses the findings of this early consultation may enhance feelings of ownership—and hence adoption—of those who may be asked to use the standardised system in practice.^26 45 54^

### Need for evidence-based, co-designed implementation strategies

Our study indicates that an important next step is to co-design—with relevant professionals and service users—clinical tools and other supporting resources for use of the proposed approach in practice.^25 26 39 68^ Agreed intrapartum practice tools, informed by co-design processes and human factors engineering principles,^26^ will likely aid standardisation across maternity services when combined with multiprofessional training.^39 68^ Standardisation will also be aided by new national guidelines that include the latest evidence and clinical agreement on systematically assessing a set of intrapartum risk factors alongside other fetal heart rate features.^10 16 17^ These new guidelines were informed by our findings and have already started to address the varying views on clinical definitions and thresholds for actions identified in our study.^10^

Another important step towards standardisation is the co-design of high-quality implementation strategies.^46 49 54 69^ This should start with consultation with senior stakeholders and frontline staff on the optimal delivery system to identify organisational barriers to improvement and potential ways to address these barriers.^69 70^ Implementation support will likely need to differ across varying types of units, such as more and tailored support for units with limited capability to improve.^69 71 72^ Further key areas will be the strengthening of clinical governance systems,^73^ and evaluation mechanisms to identify learning and areas of attention when national standardisation of the proposed approach is implemented.^59 74 75^ Critical to the implementation approach will be the use of deimplementation strategies for local practices^76^ and a need for considering adaptive strategies when services are under pressure.^65^,^67^ Participants themselves offered several important suggestions for what might help in achieving these goals, including highlighting the evidence base, emphasising the potential for improved clinical outcomes, retaining a role for clinical judgement and local adaptations where needed and ensuring that the approach is practical and highly usable.

Finally, participants highlighted the importance of considering service users’ concerns during fetal monitoring, which aligns with the imperative to include patient/family concern in track-and-trigger systems.^23^ They also expressed a need for support in this area, such as for effective communication and decision-making with those in labour and their birth partners, in accordance with urgent calls for improvement in UK maternity care.^3 24^ These aspects should be strongly grounded and addressed in a new standardised approach to detecting and responding to fetal deterioration^26^ and associated multiprofessional training and implementation support for the approach.^27 28 68^

### Strengths and limitations of this study

These surveys are the first to explore the range of views of maternity professionals on detecting and responding to intrapartum fetal deterioration, with strong potential to inform co-design and implementation of a standardised approach as part of the ABC programme.^27 28 68^ The findings illustrate the importance of early engagement of those intended to use a new standardised approach in practice. Methodological strengths of this study include the use of a multi-disciplinary approach in devising the surveys and the use of an online collaboration platform that enabled wide and user-friendly participation.

While these surveys are the largest on this topic to date, the sample is limited to the UK only. Views of professionals in other countries may be different. The wide participation of various professionals across the UK—recruited through a range of stakeholder networks—is a strength, though not every UK maternity unit was represented. The sample size precluded meaningful statistical comparison of subgroups, limiting interpretation of comparisons between different professional groups. It is possible that our use of complete-case analysis may have introduced some biased estimates if there were differential responses to particular questionnaire items.^77^

## Conclusions

This study shows strong professional support for the principle of a standardised approach that systematically considers a range of risk factors alongside fetal heart rate features to support detection of intrapartum fetal deterioration. The findings highlight the widely varying views on thresholds for action to the risk factors and attitudes towards national and international guidelines, likely caused by a field in which maternity professionals navigate sociotechnical complexities without clear consensus on thresholds for action. The findings also demonstrate that more definitional clarity is needed on some of these risk factors, and that evidence and agreement need to be built on thresholds for action. Such clarity and agreement may help ease the tension between standardisation and preference for locally established approaches. The study highlights the importance of co-design processes for development and implementation of proposed changes in clinical practice, including the key role of early engagement with professionals.

## supplementary material

10.1136/bmjopen-2024-084578 online supplemental file 1

10.1136/bmjopen-2024-084578 online supplemental file 2

## Data Availability

Data are available on reasonable request.
