# Supplementary material for: Detecting and responding to deterioration of a baby during labour: surveys of maternity professionals to inform co-design and implementation of a new standardised approach
Source: BMJ Open. 2025 Mar 6;15(3):e084578. doi: 10.1136/bmjopen-2024-084578 (PMC11887309; doi:10.1136/bmjopen-2024-084578)
Supplement: online supplemental file 1 [file bmjopen-15-3-s001.pdf]

Supplement 1 – Questions used in survey 1

About you and your unit

Before we start, can we confirm we have the correct information about your role? If you previously gave us this information, you will find the answers pre-populated below. If your answers have changed or you did not provide this information before, please feel free to add or amend as necessary.

- What is your professional role?
    - Midwife band 5 – 7
    - Midwife band 8 – 9
    - Consultant Obstetrician
    - Trainee Obstetrician (including registrars and trust grade doctors)
    - Consultant Anaesthetist
    - Trainee Anaesthetist (including registrars and trust grade doctors)
    - MSW (Maternity Support Worker)
    - HCA (Health Care Assistant)
    - Student Midwife
    - Other, please specify

- Do you hold any clinical leadership roles?
    - Yes
    - No

- What region in the UK do you work?
    - East of England
    - London
    - Midlands
    - North East and Yorkshire
    - North West
    - Northern Ireland
    - Scotland
    - South East
    - South West
    - Wales

- What type of maternity unit or setting do you currently work in? If you work in more than one setting, please select all options that apply.
    - Obstetric unit
    - Alongside midwifery unit (a midwifery-led unit or birth centre situated in the same hospital or on the same site as an obstetric unit)
    - Freestanding midwifery unit (a midwifery-led unit or birth centre not situated in a hospital or site with an obstetric unit)
    - Community
    - Other [please write in]

- It would be really helpful if you could provide the name of the Trust/s you are employed by, if applicable: …………………… [free-text box]

Script of introductory video

Please watch the introductory video below.

	**Script/voiceover**
Visual 1	Preventable harm during labor can be catastrophic.
Visual 2	Everyone is committed to reducing avoidable injury to babies.
Visual 3	Intrapartum brain injury is often linked to problems with fetal surveillance in labor.
Visual 4	Intermittent auscultation or electronic fetal monitoring can be used to monitor the baby’s heart rate.
Visual 5	But the evidence is clear: effective fetal surveillance requires a whole systems approach.
Visual 6	Instead of asking ‘Is the baby’s heart rate OK?’ a better approach is to ask, ‘Is the baby OK?’
Visual 7	The Royal College of Midwives and the Royal College of Obstetricians and Gynaecologists have been working together over the last two years to outline a collaborative approach to fetal surveillance. It is one that will support, not replace, clinical judgement. Its key aim is to ‘make the right way the easy way’.
Visual 8	We want to work together to get this right. We’d love you to join us.

We will now ask you **five questions** about your views on a collaborative approach to fetal surveillance. Your responses will help us explore how best to refine the outline approach, and will inform future development activities including usability testing.The activity should take no longer than **15 minutes** to complete. For each question you have the option of providing additional comments. You can also provide feedback at the end of the survey.

Question 1. To what extent do you agree with this statement:

I support a personalised approach to fetal surveillance that includes monitoring the fetal heart rate (FHR) as *one* – but not the only one – of a range of indicators of potential fetal deterioration.

Strongly disagree	Disagree	Neither agree nor disagree	Agree	Strongly agree

Please provide additional comments (optional)

Why are we asking this? Please click here: *[pop-up box]*

“Current research evidence on the risk factors for intrapartum hypoxia is clear that fetal heart rate is only one of a range of indicators of the potential for hypoxia. A more personalised approach has been recommended by the RCOG/RCM task and finish group on fetal surveillance, taking account of a wider range of factors, such as maternal pyrexia and the presence of meconium.”

Question 2: To what extent do you agree with this statement:

A national standardized approach to fetal surveillance, used across all maternity settings, would benefit the safety and quality of intrapartum care.

Strongly disagree	Disagree	Neither agree nor disagree	Agree	Strongly agree

Please provide additional comments (optional)

Why are we asking this? Please click here: *[pop-up box]*

“Consensus within the RCOG/RCM task-and-finish group on fetal surveillance was that a national standardized approach has the potential to improve safety by providing a consistent framework for training and practice of intrapartum fetal surveillance”

Question 3: Research evidence shows that there are certain key indicators of potential intrapartum fetal deterioration. You will find these below.

How important do you think these indicators are in practice when assessing potential intrapartum fetal deterioration?

	Not at all important	Slightly important	Moderately important	Very important	Extremely important
Changes in fetal heart rate (for IA) and changes in CTG (for EFM)					
Significant meconium-stained liquor					
Oxytocin augmentation					
Delay in progress in labor					
Maternal pyrexia (38°C or above on a single reading, or above 37.5^{o}C on two consecutive occasions one hour apart)					
Diagnosed or suspected fetal growth restriction or small for gestational age (less than the 10th centile					
Intrapartum vaginal bleeding					
Hyperstimulation (>4:10)					

Please provide additional comments (optional)

Why are we asking this? Please click here: *[pop-up box]*

The RCOG/RCM task and finish group recommend using these indicators as part of a personalised approach to assessing potential fetal deterioration, based on current research evidence. Asking you about how important you think these indicators are is crucial to developing clinical tools and implementing an approach that works well in practice.

Question 4: Getting the right wording to describe clinical indicators is not always easy. Do you think the current descriptions for these indicators need to be improved? [Yes/No]

	Yes	No
Changes in fetal heart rate (for IA) and changes in CTG (for EFM)		
Significant meconium-stained liquor		
Oxytocin augmentation		
Delay in progress in labor		
Maternal pyrexia (38°C or above on a single reading, or above 37.5^{o}C on two consecutive occasions one hour apart)		
Diagnosed or suspected fetal growth restriction or small for gestational age (less than the 10th centile		
Intrapartum vaginal bleeding		
Hyperstimulation (>4:10)		

Please provide additional comments (optional)

Why are we asking this? Please click here: ***[pop up box]***

Descriptions for these indicators have come from a variety of sources, including national guidelines. We would like to know whether you think any of these descriptions could be improved, for example by better clarifying their meaning or the thresholds they use.
