# Supplementary material for: Detecting and responding to deterioration of a baby during labour: surveys of maternity professionals to inform co-design and implementation of a new standardised approach
Source: BMJ Open. 2025 Mar 6;15(3):e084578. doi: 10.1136/bmjopen-2024-084578 (PMC11887309; doi:10.1136/bmjopen-2024-084578)
Supplement: online supplemental file 2 [file bmjopen-15-3-s002.pdf]

Supplement 2 – Questions used in survey 2

About you and your unit

Before we start, can we confirm we have the correct information about your role? If you previously gave us this information, you will find the answers pre-populated below. If your answers have changed or you did not provide this information before, please feel free to add or amend as necessary.

- What is your professional role?
    - Midwife band 5 – 7
    - Midwife band 8 – 9
    - Consultant Obstetrician
    - Trainee Obstetrician (including registrars and trust grade doctors)
    - Consultant Anaesthetist
    - Trainee Anaesthetist (including registrars and trust grade doctors)
    - MSW (Maternity Support Worker)
    - HCA (Health Care Assistant)
    - Student Midwife
    - Other, please specify

- Do you hold any clinical leadership roles?
    - Yes
    - No

- What region in the UK do you work?
    - East of England
    - London
    - Midlands
    - North East and Yorkshire
    - North West
    - Northern Ireland
    - Scotland
    - South East
    - South West
    - Wales

- What type of maternity unit or setting do you currently work in? If you work in more than one setting, please select all options that apply.
    - Obstetric unit
    - Alongside midwifery unit (a midwifery-led unit or birth centre situated in the same hospital or on the same site as an obstetric unit)
    - Freestanding midwifery unit (a midwifery-led unit or birth centre not situated in a hospital or site with an obstetric unit)
    - Community
    - Other [please write in]

- It would be really helpful if you could provide the name of the Trust/s you are employed by, if applicable: …………………… [free-text box]

Background

The Avoiding Brain Injury in Childbirth (ABC) collaboration is developing a new holistic approach to fetal wellbeing, one that uses fetal heart rate as one, but not the only one, of a range of risk factors to fetal wellbeing in labor.

The ABC collaboration has developed prototype tools for monitoring and recording fetal risk factors during labor that inform taking action if there is suspected fetal deterioration. However, maternity staff called for the definitions of these risk factors to be improved.

We want to hear **your views** on how best to describe the **risk factors and thresholds** that might trigger action – such as, for example, moving from intermittent auscultation to electronic fetal monitoring, or moving from a midwifery-led to an obstetric-led birth setting.

We also want to hear **your views** on developing **resources to support good practice** when there are concerns about a baby during labor.

Please answer the series of questions in this survey using **your own clinical judgement**, not that of your current trust guidelines.

The activity should take no longer than **15 minutes** to complete.

Meconium

What language should be used to describe meconium if you feel it represents a **low risk** to the baby? Please tick the one that you think is most applicable.

- ☐ Thin
- ☐ Grade 1
- ☐ Insignificant
- ☐ None of the above – I do not think there is a need to differentiate between types of meconium
- ☐ Other, please state: [free text box]

What language should be used to describe meconium if you feel it represents a **high risk** to the baby? Please tick the one that you think is most applicable.

- ☐ Thick
- ☐ Grade 2/3
- ☐ Significant
- ☐ None of the above – I do not think there is a need to differentiate between types of meconium
- ☐ Other, please state: [free text box]

During a labor in a low risk setting with the use of intermittent auscultation, a midwife establishes presence of meconium. The midwife might refer to this as “thin meconium” or “insignificant meconium”. At which point should the person in labor be transferred from the low-risk setting to obstetric-led care?

- ☐ The presence of ANY meconium should prompt advising or offering immediate transfer, regardless of any other risk factors

OR please tick all of the following that may apply:

- ☐ In the presence of slow progress

☐ In the presence of maternal pyrexia

☐ If there are fetal heart rate concerns

☐ If the pregnancy is 39 weeks or less

☐ If it is a new appearance of meconium during labor

Delay in progress of labor

Do you use the NICE definitions of delay in labor? [insert link to pop-up box]

☐ Yes

☐ No

☐ Not sure

POP UP BOX: NICE DEFINITIONS OF DELAY IN LABOUR

Definition of delay in first stage

- 'If delay in the established first stage is suspected, assess all aspects of progress in labour when diagnosing delay, including: **• cervical dilatation of less than 2 cm in 4 hours for first labours • cervical dilatation of less than 2 cm in 4 hours or a slowing in the progress of labour for second or subsequent labours** • descent and rotation of the baby's head • changes in the strength, duration and frequency of uterine contractions.

Definition of delay in second stage:

- **For a nulliparous woman:** 'birth would be expected to take place within 3 hours of the start of the active second stage in most women **• diagnose delay in the active second stage when it has lasted 2 hours'**; 'suspect delay if progress (in terms of rotation and/or descent of the presenting part) is inadequate after 1 hour of active second stage.'
- **For a multiparous woman:** 'birth would be expected to take place within 2 hours of the start of the active second stage in most women'; **'diagnose delay in the active second stage when it has lasted 1 hour.' '**suspect delay if progress (in terms of rotation and/or descent of the presenting part) is inadequate after 30 minutes of active second stage.'

Do you think the NICE definitions of delay in labor are in need of improvement? [insert link to pop-up box]

☐ Yes

☐ No

☐ Not sure

Please add suggestions in the box below if you think the NICE definitions of delay in labor can be improved.

[Free text box]

Vaginal bleeding

At what point do you think, during labor, a blood-stained show should be classified as intrapartum vaginal bleeding, indicating a need for action to be taken (e.g. escalate risk factors to MDT, transfer to obstetric-led care)? Please tick all that apply.

☐ When it is greater than 50 ml

☐ When it occurs immediately after vaginal examination

☐ When there is no mucous present

☐ When it presents as fresh vaginal bleeding

☐ When there are heavily soaked pads

☐ Other, please describe: [free text]

Contractions

At what point would you consider there to be too many contractions, with a need for action to be taken (e.g. reduction in oxytocin, escalate risk factors to MDT, transfer to obstetric-led care)? Please tick all that apply.

☐ 3 to 4 contractions in 10 minutes

☐ 4 contractions in 10 minutes

☐ 4 to 5 contractions in 10 minutes

☐ 5 or more contractions in 10 minutes

☐ I don't think any of the above require action

Which other features would you also take into account when thinking about contractions as an intrapartum risk factor? Please tick all that apply.

☐ resting tone

☐ palpated strength of contraction

☐ length of each contraction

☐ use of oxytocin

☐ I would not take any of the above into account

Maternal pyrexia in labor

At what point does action need to be taken in relation to maternal pyrexia in labor (e.g. escalate risk factors to MDT, transfer to obstetric-led care)? Please choose one of the following options:

☐ Maternal pyrexia defined as 38°C or above on a single reading or 37.5°C or above on two consecutive readings **one hour** apart

☐ Maternal pyrexia defined as 38°C or above on a single reading or 37.5°C or above on two consecutive readings **two hours** apart

☐ Any two non-consecutive readings of 37.5°C or above

☐ Any one reading of 37.5°C or above

☐ Other, please describe: [free text]

Fetal heart rate changes

If you heard a deceleration on intermittent auscultation, what action would you take? Please choose **one** of the following options:

☐ Continue with usual labor care

☐ Increase the frequency of auscultation, listening immediately following the next three contractions to assess for further decelerations

☐ Transfer to obstetric-led care

☐ Other, please describe: [free text]

Thinking about changes in the CTG as an intrapartum risk factor, how would you express a significant rise in the baseline above initial baseline rate at start of labor? Please choose **one** of the following options:

☐ Beats per minute (bpm): 10 bpm

☐ Beats per minute (bpm): 15 bpm

☐ Beats per minute (bpm): 20 bpm

☐ Beats per minute (bpm): Other, please describe: [free text]

☐ Percentage rise from initial baseline: 10%

☐ Percentage rise from initial baseline: 15%

☐ Percentage rise from initial baseline: 20%

☐ Percentage rise from initial baseline: Other, please describe: [free text]

Thinking about changes in the CTG as an intrapartum risk factor, how would you express a significant period of reduced variability? Please choose **one** of the following options:

☐ Any period of reduced variability

☐ Less than 30 minutes

☐ 30 to 50 minutes

☐ 50 to 90 minutes

☐ More than 90 minutes

☐ Other, please describe: [free text]

If intrapartum risk factors such as meconium or sepsis are present, at what point would the variability concern you? Please choose **one** of the following options:

☐ Any period of reduced variability

☐ Less than 30 minutes

☐ 30 to 50 minutes

☐ 50 to 90 minutes

☐ More than 90 minutes

☐ Other, please describe: [free text]

When considering increased variability (bandwidth of >25), what period of time should prompt further action? Please choose **one** of the following options:

- ☐ Any period of increased variability
- ☐ Less than 5 minutes
- ☐ 5 to 10 minutes
- ☐ 11-15 minutes
- ☐ 16-20 minutes
- ☐ 21-25 minutes
- ☐ 26-30 minutes
- ☐ More than 30 minutes
- ☐ Other, please describe: [free text]

How do you classify prolonged decelerations/bradycardia?

- ☐ More than 5 minutes
- ☐ More than 3 minutes
- ☐ Other, please describe: [free text]

Which types of decelerations would concern you **and** prompt you to re-examine the full clinical picture?

- ☐ Decelerations during 50% or more of contractions ("repetitive")
- ☐ Decelerations during less than 50% of contractions ("intermittent" or "non-repetitive")
- ☐ Decelerations lasting 60 seconds or more
- ☐ Decelerations lasting less than 60 seconds
- ☐ V shaped deceleration(s)
- ☐ Late deceleration(s)
- ☐ Slow return to baseline after a contraction
- ☐ Fetal heart rate spends more time decelerating at the baseline

Guidelines for fetal heart rate monitoring

Which guideline/s for electronic fetal monitoring does your place of work currently use? Please choose **one** of the following options:

- ☐ NICE
- ☐ FIGO
- ☐ Physiological
- ☐ NICE and FIGO
- ☐ NICE and Physiological
- ☐ FIGO and Physiological
- ☐ Not applicable
- ☐ Other (please describe)

Which guideline/s for electronic fetal monitoring would you use by preference? Please choose **one** of the following options:

- ☐ NICE
- ☐ FIGO
- ☐ Physiological
- ☐ NICE and FIGO
- ☐ NICE and Physiological
- ☐ FIGO and Physiological
- ☐ Not applicable
- ☐ Other (please describe)

Please give any **additional comments** you may have on intrapartum risk factors such as meconium, delay in labor, bleeding, contractions, pyrexia or fetal heart changes.

[Free text box]

It's important that the ABC tool reflects a focus on fetal well-being and uses language that is accessible and acceptable to staff, those in labor and their partners. The working name for the ABC tool is currently "ABC Intrapartum fetal surveillance tool" but other options can be explored. What would you recommend?

- ☐ I recommend sticking with "ABC intrapartum fetal surveillance tool".
- ☐ I recommend finding a new name for the tool.

If you have a suggestion for the new name, please write it below.

[Free text box]

What term should be used to describe the parameters used for monitoring and recording fetal wellbeing during labor:

- ☐ Risk factors (e.g. fetal risk factors, intrapartum risk factors, start of labor risk factors)
- ☐ Clinical indicators (e.g. intrapartum clinical indicators)
- ☐ Fetal wellbeing risk factors
- ☐ Fetal wellbeing indicators
- ☐ Other, please specify: [free text box]

Resources to support good practice

The ABC programme is a whole-system approach. The associated tools to detect and respond to suspected fetal deterioration need to be supported by good practice when there are concerns about a baby during labor. How useful would you find summary infographics and links to further resources on the following:

	Not at all useful	Somewhat useful	Useful	Very useful	Extremely useful
Features of safe maternity units					
How to gain professional confidence in the new tools					
Effective teamwork to support detection and response to suspected fetal deterioration					
Situational awareness					
When and how to escalate in different situations					
Effective communication and decision-making with those in labor and their partners					
How to create a supportive culture					
How to deal with disagreements					

Would you be interested in helping to develop resources to support escalation and other good practice when there are concerns about a baby during labor? Please tick one or more of the following options:

1. Yes, I would be interested in taking part in a short interview
2. Yes, I would be interested in taking part in an online workshop
3. Yes, I would be interested in commenting on drafts by email
4. No, I would not be interested
