# Supplementary material for: Detecting and responding to deterioration of a baby during labour: surveys of maternity professionals to inform co-design and implementation of a new standardised approach
Source: BMJ Open. 2025 Mar 6;15(3):e084578. doi: 10.1136/bmjopen-2024-084578 (PMC11887309; doi:10.1136/bmjopen-2024-084578)
Supplement: Supplementary file 3 [file bmjopen-2024-084578.reviewer_comments.pdf]

PEER REVIEW HISTORY

BMJ Open publishes all reviews undertaken for accepted manuscripts. Reviewers are asked to complete a checklist review form and are provided with free text boxes to elaborate on their assessment. These free text comments are reproduced below.

ARTICLE DETAILS

Title (Provisional)

Detecting and responding to deterioration of a baby during labour: surveys of maternity professionals to inform co-design and implementation of a new standardised approach

Authors

van der Scheer, Jan; Blott, Margaret; Dixon-Woods, Mary; Olsson, Annabelle; Moxey, Jordan; Kelly, Sarah; Woodward, Matthew; Maistrello, Giulia; Randall, Wendy; Blackwell, Sarah; Hughes, Chloe; Walker, Caroline; Dewick, Louise; Bahl, Rachna; Draycott, Tim J; Thiscovery Authorship Group, Thiscovery Authorship Group; ABC Contributor Group, ABC Contributor Group; Burt, Jenni

---

VERSION 1 - REVIEW

**Reviewer**	**1**
**Name**	**Evans, Mark I.**
**Affiliation**	**Icahn School of Medicine at Mount Sinai**
**Date**	**22-Mar-2024**
**COI**	**This reviewer has published on a quantitative method for interpretation of CTG.**

This is a survey of hundreds of British stakeholders in the use of fetal monitoring asking the question of whether or not standardization of the interpretation would be useful. Of course the answer was yes, but the survey did little more than articulate the glaring need for such standardization as the responses were so varied to the point of demonstrating why they are so desperately needed.

1. As a study, this does little to advance improvements other than helping to establish what has been obvious to experts in the field for decades.

2. From a political point, it may be necessary to convince "everyone" of the need for such standardization. As such the major conclusion of the study would be better articulated as

a. The discrepancy among providers is enormous.

b. Such explains part of the poor performance of monitoring as practiced.

c. Such further explains why an increasing number of patients/providers are moving. away from continuous monitoring which was more prevalent in the past.

3. It would be useful for the authors to at least propose what types of standardization they are suggesting more than just a couple of sentences. There is literature on this topic.

**Reviewer**	**2**
**Name**	**Olsen, Ole**
**Affiliation**	**University of Copenhagen**
**Date**	**18-Apr-2024**
**COI**	**None**

As the input box for my comments does not retain bold, italics, etc in my written text, I have split my response into three attachments to be read in the following order:

1. Comments to the author(s) and the editor

2. Comments to the editor

3. Comments solely to the author(s)

As the option to designate the attached file into two options was not visible until the first file had been uploaded, and only two options were possible, I decided to designate my last attachment as"Author & Editor". I am sure you will be able to manage anyway :-)

Sincerely

Ole Olsen

ad 2. Is the abstract accurate, balanced and complete? - No

When I accepted the invitation to review the paper, I looked forward to dive into the emerging evidence that according to the abstract motivated the focus of and the investigations described in the paper. I did so particularly because the fathers of EBM at the outset wrote “Evidence based medicine is not "cookbook" medicine”. Thus finding evidence that suggests that diagnosis treatment of specific conditions is likely to benefit from a standardised approach would be interesting. Unfortunately, however, I was disappointed. Reading the introduction, I could not immediately see that the claim in the abstract was substantiated. I thus searched for the words “emerging” and “evidence” but did not find them. However, the words “likely to benefit from a standardised approach …” from the abstract did re-appear but this time not with reference to evidence but rather with refence to two papers, one (reference 8) - though it was to a “summary and update of NICE guidance” - had all (or at least the first ten) recommendations described with the words “Based on the experience and opinion of the Guideline Committee (GC)”, the other reference was specifically about consensus and not evidence (ref 11). However, the next two references on the reference list (ref 12-13) were to systematic reviews that might offer

some empirical evidence. Unfortunately, however, upon looking into them they do not seem to support the claim made in the very first sentence of the abstract. On the contrary. Reference 12 states (my emphasis):

> **Conclusion:** There are several fundamental methodological limitations in the PTTT literature, and the predominance of single-site studies carried out in specialist centres greatly limits generalisability. With limited evidence of effectiveness, calls to make PTTT mandatory across all paediatric units are not supported by the evidence base.

Reference 13 states:

> AUTHORS' CONCLUSIONS: Use of TTS/EWS in maternity care … may result in little to no difference in neonatal death. Both of the included studies were conducted in low-resource settings where the use of TTS/EWS might potentially confer a different effect to TTS/EWS use in high-resource settings. Further high-quality trials in high- and middle-resource settings, as well as in discrete populations of high- and low-risk women, are required.

The litte evidence there is ("little to no difference in neonatal death") is even in the opposite direction: OR 1.06, 95% CI 0.62 to 1.84.

Now, if it is not the claim made in the abstract that motivated the research project, what did then motivate the project? Is there some sort of elephant in the room? I will address this issue in the document Comments solely to the author(s).

Comments solely to the author(s)

The very first six references were all listed in relation to one sweeping statement covering everything from a very local level to the global level. My eyes were catched by the second reference to a particular report covering East Kent. It seemed as if a terribly and utterly catastrophic mismanagement in a specific health care district that had been ongoing for more than a decade despite several attempts to solve was the real motivation for the whole project. My guess from my many conversations with Danish midwives (I am a statistician myself) for more than thirty years would suggest that "systematically considering fetal heart rate changes alongside other developing risk factors during labour" is a core part of midwives' clinical expertise and that the various developing risk factors would always be judged as part of the entirety of the birthing woman, the signs, the context and the developing situation at large, i.e. not "cookbook" medicine.

Nevertheless it seemed as if more than a dozen co-authors had the idea that a new standardised approach (based on opinion and consensus rather than on evidence) would solve the problem - the problem that up to a dozen attempts to intervene in the ongoing mismanagement at least in East Kent by making part of maternity care to "cookbook" medicine could not solve - would solve the problem. And on closer reading it seemed as if the solution to the local problem of mismanagement had already been resolved at a national level by means of consensus, as the core question in the survey had the following accompanying text (my emphasis):

> Why are we asking this? Please click here: [pop-up box]

> "Consensus within the RCOG/RCM task-and-finish group on fetal surveillance was that a
> national standardized approach has the potential to improve safety by providing a consistent
> framework for training and practice of intrapartum fetal surveillance"

Thus it is not a great surprise that the members of the two organisations in a (ten minute?) survey would agree to this consensus. The entire paper thus reminds me of a self fulfilling prophecy or circular reasoning as. This is also is reflected in the abstract where the phrase "a standardised approach" appears prominently three times:

> ABSTRACT:
> **Objectives**: Emerging evidence suggests that detecting and responding to deterioration of a baby during labour is likely to benefit from a standardised approach …
> **Results**: Over 90% of participants supported the principle of a standardised approach …
> **Conclusions**: We found strong professional support for the principle of a standardised approach …

Most readers only read the abstract. The question is: What is left out here?

The very last sentence of the long result section in the abstract reads: "Over 75% of participants agreed on the usefulness of a range of resources to support good practice when implementing the approach", but there is no mention of any of these specific practices. In the main txt the following text occurs:

> - how to create a supportive culture (n=271, 79%),
> - effective teamwork to support detection and response to possible fetal deterioration (n=266, 78%),
> - situational awareness (n=263, 77%),
> - how to deal with disagreements (n=260, 76%), and
> - effective communication and decision-making with those in labour and their partners (n=256, 75%)

It is not obvious to me why these highly desired good practices are not mentioned in the abstract. Given that the survey seems to be designed to get support for a standardised approach, the support for these other approaches seem very high (and the suppport could perhaps even have surpassed standardised approach had the survey had a less biased approach).

Furthermore, when I searched reference 8 for the emergent evidence for a standardized approach mentioned in the very first line of the abstract, the word 'standardized' did not occcur. But while typing I saw by chance that the word 'standard' did occur once (in the section on Implementation). It appeared along with the sentence: "Another potential barrier to successful implementation is the current national shortage of midwives and doctors". This aspect is completely left out in your paper. If there is a national shortage of midwives and doctors, the core clinical skills asked for above may be lost in the educational system. I

doubt that some infographics as mentioned in Survey 2 can substitute for a well organised educational system.

Finally, lack of evidence and fear of litigation is mentioned in a quote (that is not further followed up):

> *It is really difficult to ascertain what is the best thing to do is, when clinical research on*
> *each of the areas is so limiting. The fear of litigation is high [...].” (Midwife)*

It is hard for my to imagine that it is helpful to leave out bullying, poor management, shortage of midwives, fear of litigation, and lack of transfer of basic clinical skills from the experecned to the younger and only focus on a written standardized approach as a possible solution in relation to detecting and responding to deterioration of a baby during labour.

In additions to my concerns above related to the internal logic of the paper, I googled “crisis in uk maternity care” and got lots of relevant hits, e.g.

> Almost two-thirds of maternity units provide dangerously substandard care that puts women and babies at risk, the NHS watchdog has said – The Guardian
> Midwives are being driven out of the NHS by understaffing and fears they can't deliver safe care to women in the current system, according to a new survey – ICM

One of the hits mention paper work:

> increase in paperwork due to the demands of organisational reporting and accounting (for both internal and external purposes)” has “resulted in less and less time for midwives to spend with women in labour and during birth.” “The increasing fear of litigation in the maternity services has also increased the general sense that spending time on record-keeping is a critical activity for both midwives and doctors, since these records are the basis for defence if there is such a case.”
> https://www.equaltimes.org/a-perfect-storm-why-uk-midwives

The challenges seem to be similar in other high income countries (Olsen & Clausen 2023):

> Midwives have increasingly been leaving the profession (Curtis 2006; Lind 2021; Svensson 2021), describing the situation at the hospital as “war like” (Geraghty 2019), or describing themselves as working “in a highly strained, factory-like, over-medicalised context with high demands and lack of organisational resources and support systems” (Hansson 2020), issues that have led to a high turnover, with more than one-third of all newly educated midwives leaving the labour ward within four to five years (Lind 2021), and midwifery students stating that “Current maternity care is medicalized and managed from a pathological point of view. Midwives flee maternity care because they are not allowed to be midwives”(Svensson 2021)

Central fetal monitoring is not mentioned in the paper. Kirsten Small et al have written a series of papers on this. In one it is stated that

Findings: The introduction of the central monitoring system was associated with clinical decision making without complete clinical information. Midwives' work was disrupted. …. Midwives described a shift in focus away from the birthing woman towards documenting in the central monitoring system.
https://pubmed.ncbi.nlm.nih.gov/34092530/

I do not know if central fetal monitoring is in widespread use in the UK, it is not mentioned in the paper. If it is, it might be a relevant aspect to include.

The projects would have gained from a more open-minded involvement of stake holders instead of just aiming for support (and input) for an already chosen solution.

I am sorry that my review of your project is fairly negative. However, I hope you can use some of my feed-back anyway.

**Reviewer**	**3**
**Name**	**Phillips, Kerry**
**Affiliation**	**Cardiff University**
**Date**	**25-Oct-2024**
**COI**	**None**

Page 6, lines 53 - 56, mentions PPI. However, the citation here is misleading; this study did not involve service users.

Research into the consistency of approach to the deterioration of a baby's condition during labour is laudable and much needed. However, the language in this draft paper tends to disembody the pregnant woman or birthing person. I would suggest that within the introduction, the authors signal the importance of autonomy and agency and the need for clinicians to meaningfully discuss their concerns about a deteriorating picture during labour with those in labour and their people.

VERSION 1 - AUTHOR RESPONSE

ID	Comments to the authors	Authors' response
	**Reviewer 1**	

R1-1	This is a survey of hundreds of British stakeholders in the use of fetal monitoring asking the question of whether or not standardization of the interpretation would be useful. Of course the answer was yes, but the survey did little more than articulate the glaring need for such standardization as the responses were so varied to the point of demonstrating why they are so desperately needed. As a study, this does little to advance improvements other than helping to establish what has been obvious to experts in the field for decades.	Thank you for your review of the paper.<br><br>As we outline in the introduction, a lack of engagement and co-design with frontline staff is likely one of the reasons why improvement and standardisation of the detection and response to intrapartum fetal deterioration across maternity services has been unsuccessful, notwithstanding what may have been obvious to experts in the field. This is an area characterised by proliferation of local approaches in practice. The surveys presented in our paper are a first step towards addressing this problem. Further steps of this co-design process are presented in other publications cited in the paper (e.g. https://qualitysafety.bmj.com/content/33/4/258).<br><br>Furthermore, our surveys of those working at the frontline of care are notable for finding that, despite the sense of local ownership people may feel about their own approach, 90% of participants supported the principle of a standardised approach based on a track-and-trigger system principles. This is not an outcome that could have been predicted in advance.<br><br>The surveys are also important in identifying some of the challenges of standardisation in this area, providing key information to support sound implementation.<br><br>The value of our findings is demonstrated in their influence on NICE guidance on clinical definitions and thresholds for action (https://www.bmj.com/content/379/bmj.o2854.long).

R1-2	From a political point, it may be necessary to convince "everyone" of the need for such standardization. As such the major conclusion of the study would be better articulated as a. The discrepancy among providers is enormous.<br>b. Such explains part of the poor performance of monitoring as practiced.<br>c. Such further explains why an increasing number of patients/providers are moving away from continuous monitoring which was more prevalent in the past.	We have revised the conclusion to further highlight our findings on the widely varying views on thresholds for action during intrapartum fetal monitoring, including its potential causes. Our study did not identify evidence that women and maternity staff are moving away from continuous fetal monitoring because of variation between services in detecting and responding to intrapartum fetal deterioration.

R1-3	It would be useful for the authors to at least propose what types of standardization they are suggesting more than just a couple of sentences. There is literature on this topic.	We have included further detailin the discussion, including how our study has informed a national programme to support standardisation of detection and response to intrapartum fetal deterioration (see revised introduction and discussion re: Avoiding Brain Injury in Childbirth [ABC] programme).
	**Reviewer 2**	
R2-1	The very first six references were all listed in relation to one sweeping statement covering everything from a very local level to the global level. My eyes were catched by the second reference to a particular report covering East Kent. It seemed as if a terribly and utterly catastrophic mismanagement in a specific health care district that had been ongoing for more than a decade despite several attempts to solve was the real motivation for the whole project. My guess from my many conversations with Danish midwives (I am a statistician myself) for more than thirty years would suggest that "systematically considering fetal heart rate changes alongside other developing risk factors during labour" is a core part of midwives' clinical expertise and that the various developing risk factors would always be judged as part of the entirety of the birthing woman, the signs, the context and the developing situation at large, i.e. not "cookbook" medicine.	Thank you for your review of the paper.<br><br>Our study was not motivated by the East Kent report; we were referencing that report as an example of some of the problems in UK maternity care (see our response to R2-2 below). The study has informed a national programme to support standardisation of detection and response to intrapartum fetal deterioration (see revised introduction and discussion re: Avoiding Brain Injury in Childbirth [ABC] programme).<br><br>Failures in accurate and timely recognition and response of a deteriorating baby during labour continue to be a major contributor to global incidence of hypoxic ischaemic encephalopathy (HIE). The reasons for these are likely varied, but findings in the UK clearly indicate the need for a more systematic focus on risk factors beyond fetal heart rate features. Although the scientific literature is clear about the need to consider multiple risk factors during labour, the means by which that could be done have not previously been structured into an evidencebased standardised approach in the UK.
		We have revised the introductory sentences to clarify that failure to detect intrapartum fetal deterioration is a consistent and major problem across UK maternity care, and that this is linked to a tendency to over-focus solely on fetal heart rate features rather than a more holistic assessment of intrapartum risk factors. We have also explained that the National Institute for Health and Care Excellence (NICE) has recommended this too.

R2-2	Nevertheless it seemed as if more than a dozen co-authors had the idea that a new standardised approach (based on opinion and consensus rather than on evidence) would solve the problem - the problem that up to a dozen attempts to intervene in the ongoing mismanagement at least in East Kent by making part of maternity care to "cookbook" medicine could not solve - would solve the problem. And on closer reading it seemed as if the solution to the local problem of mismanagement had already been resolved at a national level by means of consensus, as the core question in the survey had the following accompanying text (my emphasis):<br>*Why are we asking this? Please click here: [pop-up box]*<br>*"Consensus within the RCOG/RCM task-and-finish group on fetal surveillance was that a national standardized approach has the potential to improve safety by providing a consistent framework for training and practice of intrapartum fetal surveillance"* Thus it is not a great surprise that the members of the two organisations in a (ten minute?) survey would agree to this consensus. The entire paper thus reminds me of a self fulfilling prophecy or circular reasoning as. This is also is reflected in the abstract where the phrase "a standardised approach" appears prominently three times:<br>*ABSTRACT:*<br>*Objectives: Emerging evidence suggests that detecting and responding to deterioration of a baby during labour is likely to benefit from a standardised approach*<br>*…*<br>*Results: Over 90% of participants supported the principle of a standardised approach*<br>*…*	Recent NICE guidance has identified the need for holistic assessment of intrapartum risk factors to assess fetal wellbeing during labour. Our introduction explains how this could be supported by principles of a trackand-trigger system, including the combination of clinical judgement, listening to the concerns of the person in labour and assessing all relevant risk factors in a systematic manner. Such ways of working are currently not routinely implemented across UK maternity services, and possibly also not outside the UK either.<br><br>As per our response to R2-1 above, the study was not seeking to solve the management challenges at East Kent but to address the more general challenges of failure to detect and respond to the baby who may be deteriorating during labour (we have clarified this in the revised introduction by only referring to the East Kent and Ockenden reports as examples for the types of devastating consequences for families when fetal deterioration is inadequately detected and responded to). The need to do better in this area had been identified as a priority by multiple national reports, NHS Resolution, and an expert group, guided by review of the evidence, but the extent to which the proposed approach would be seen as acceptable and feasible to the clinical community at large could not be assessed without consultation. This is what our surveys set out to do.<br><br>As explained in the methods, the aim of the first survey was to assess support for a more standardised approach. The strong support we identified is an essential step for national implementation of such an approach. The very high support levels are also remarkable in an area plagued by a proliferation of local and differing approaches to detecting and responding to intrapartum fetal deterioration (see also our response to R1-1 above).

	Conclusions: We found strong professional support for the principle of a standardised approach …<br>Most readers only read the abstract. The question is: What is left out here?	
R2-3	The very last sentence of the long result section in the abstract reads: *"Over 75% of participants agreed on the usefulness of a range of resources to support good practice when implementing the approach", but there is no mention of any of these specific practices.*<br>In the main txt the following text occurs: *how to create a supportive culture (n=271, 79%), effective teamwork to support detection and response to possible fetal deterioration (n=266, 78%), situational awareness (n=263, 77%), how to deal with disagreements (n=260, 76%), and effective communication and decision-making with those in labour and their partners (n=256, 75%)*<br>It is not obvious to me why these highly desired good practices are not mentioned in the abstract. Given that the survey seems to be designed to get support for a standardised approach, the support for these other approaches seem very high (and the suppport could perhaps even have surpassed standardised approach had the survey had a less biased approach).	We have added some examples of findings related to the resources to support good practice to the abstract, but the abstract word count did not permit us to list the full results of these specific findings.<br>As outlined in the introduction, implementing a standardised approach at national level is very difficult. Both surveys generated important insights into facilitators for implementation – see for example the section "Suggestions for development and implementation of a standardised approach" (survey 1), and indeed the section on "Good practice resources" (survey 2).
R2-4	Furthermore, when I searched reference 8 for the emergent evidence for a standardized approach mentioned in the very first line of the abstract, the word 'standardized' did not occcur. But while typing I saw by chance that the word 'standard' did occur once (in the section on Implementation). It appeared along with the sentence: "Another potential barrier to successful implementation is the current national shortage of midwives and	We have added to the revised discussion the relevance of adaptative strategies when services are under pressure, but it was beyond the scope of this paper to describe all reasons and factors affecting optimal functioning of maternity care. The reports on maternity care referenced in the introduction provide many examples of lack of recognition and appropriate response to developing risk factors leading to avoidable intrapartum brain injury, even when adequate

	doctors". This aspect is completely left out in your paper. If there is a national shortage of midwives and doctors, the core clinical skills asked for above may be lost in the educational system. I	staffing was available. The rationale for the work presented in our paper is to address these issues. The approach needed to improve detection and response to a deteriorating baby clearly requires much more than "some infographics"

	doubt that some infographics as mentioned in Survey 2 can substitute for a well organised educational system.	and – as the introduction and discussion detail – is not the basis of what we propose.
R2-5	Finally, lack of evidence and fear of litigation is mentioned in a quote (that is not further followed up):<br>*It is really difficult to ascertain what is the best thing to do is, when clinical research on each of the areas is so limiting. The fear of litigation is high […]." (Midwife)*<br>It is hard for my to imagine that it is helpful to leave out bullying, poor management, shortage of midwives, fear of litigation, and lack of transfer of basic clinical skills from the experecned to the younger and only focus on a written standardized approach as a possible solution in relation to detecting and responding to deterioration of a baby during labour.	This paper focuses on the principles of a standardised approach, and how views from frontline staff can help shape the implementation of such an approach. It does not advocate for a written rigid standardised approach or a "cookbook" approach.<br>We have revised the discussion to include a more detailed outline of the implementation of such an approach, which requires addressing of the sociotechnical complexity of fetal monitoring and escalating concerns, including the range of issues that the reviewer highlights. It was beyond the scope of this paper to discuss all these issues in detail – excellent other publications covering these topics are referenced in the discussion.

R2-6	In additions to my concerns above related to the internal logic of the paper, I googled “crisis in uk maternity care” and got lots of relevant hits, e.g.<br>*Almost two-thirds of maternity units provide dangerously substandard care that puts women and babies at risk, the NHS watchdog has said – The Guardian*<br>*Midwives are being driven out of the NHS by understaffing and fears they can't deliver safe care to women in the current system, according to a new survey – ICM*<br>One of the hits mention paper work: *increase in paperwork due to the demands of organisational reporting and accounting (for both internal and external purposes)” has “resulted in less and less time for midwives to spend with women in labour and during birth.”*<br>*“The increasing fear of litigation in the maternity services has also increased the general sense that spending time on recordkeeping is a critical activity for both midwives and doctors, since these records are the basis for defence if there is such a case.” https://www.equaltimes.org/a-perfect-storm-why-uk-midwives* The challenges seem to be similar in other high income countries (Olsen & Clausen 2023):	Please see our response to R2-5 above. We do not think the use of newspaper articles and a review based on one trial at high risk of bias – that compared planned hospital births with planned home births in 11 participants – would strengthen the paper.

	Midwives have increasingly been leaving the profession (Curtis 2006; Lind 2021; Svensson 2021), describing the situation at the hospital as “war like” (Geraghty 2019), or describing themselves as working “in a highly strained, factory-like,over-medicalised context with high demands and lack of organisational resources and support systems” (Hansson 2020), issues that have led to a high turnover, with more than one-third of all newly educated midwives leaving the labour ward within four to five years (Lind 2021), and midwifery students stating that “Current maternity care is medicalized and managed from a pathological point of view. Midwives flee maternity care because they are not allowed to be midwives”(Svensson 2021)	

R2-7	Central fetal monitoring is not mentioned in the paper. Kirsten Small et al have written a series of papers on this. In one it is stated that<br>*Findings: The introduction of the central monitoring system was associated with clinical decision making without complete clinical information. Midwives' work was disrupted. …. Midwives described a shift in focus away from the birthing woman towards documenting in the central monitoring system.*<br>https://pubmed.ncbi.nlm.nih.gov/34092530/<br>I do not know if central fetal monitoring is in widespread use in the UK, it is not mentioned in the paper. If it is, it might be a relevant aspect to include.	Few data are available on the use and impacts on clinical decisionmaking of centralised fetal heart rate monitoring, and it was not specifically discussed by the participants in the surveys. The type of monitoring – done locally in a room or more centrally – does not affect the principles of the proposed approach.
R2-8	The projects would have gained from a more open-minded involvement of stake holders instead of just aiming for support (and input) for an already chosen solution. I am sorry that my review of your project is fairly negative. However, I hope you can use some of my feed-back anyway.	Further to our response to R2-1, R2-2 and R2-5 above, these comments seem to reflect some misunderstanding of the purpose of the paper. The aim of the surveys was not to gain support for an "already chosen" solution, but to do the following:<br>(i) assess support for a proposed standardised approach that systematically considers a range of intrapartum risk factors alongside fetal heart rate features to help detect fetal deterioration,<br>(ii) identify perceptions of the importance and clarity of a proposed set of evidence-based risk factors to underpin a standardised approach,<br>(iii) elicit views on thresholds for action for responding to suspected intrapartum fetal deterioration, and
		(iv) explore ways to support implementation of a standardised approach.

R2-9	ad 2. Is the abstract accurate, balanced and complete? - No When I accepted the invitation to review the paper, I looked forward to dive into the emerging evidence that according to the abstract motivated the focus of and the investigations described in the paper. I did so particularly because the fathers of EBM at the outset wrote “Evidence based medicine is not "cookbook" medicine”. Thus finding evidence that suggests that diagnosis treatment of specific conditions is likely to benefit from a standardised approach would be interesting. Unfortunately, however, I was disappointed. Reading the introduction, I could not immediately see that the claim in the abstract was substantiated. I thus searched for the words “emerging” and “evidence” but did not find them. However, the words “likely to benefit from a standardised approach …” from the abstract did re-appear but this time not with reference to evidence but rather with refence to two papers, one (reference 8) - though it was to a “summary and update of NICE guidance” - had all (or at least the first ten) recommendations described with the words “Based on the experience and opinion of the Guideline Committee (GC)”, the other reference was specifically about consensus and not evidence (ref 11). However, the next two references on the reference list (ref 12-13) were to systematic reviews that might offer some empirical evidence. Unfortunately, however, upon looking into them they do not seem to support the claim made in the very first sentence of the abstract. On the contrary.<br><br>Reference 12 states (my emphasis):<br>*Conclusion: There are several fundamental methodological limitations in the PTTT literature, and the predominance of singlesite studies carried out in specialist centres greatly limits generalisability. With limited evidence of effectiveness, calls to make*	The reviewer refers to international and national guidelines related to detecting intrapartum fetal deterioration. NICE guidance is based on a robust and extensive process in which all evidence and stakeholder views are weighed. Please see: https://www.nice.org.uk/about/what-we-do/ourprogrammes/nice-guidance/nice-guidelines. FIGO guidelines from the Internal Federation of Gynecology and Obstetrics are based on similar robust procedures (please see: https://www.figo.org/news/available-viewfigo-intrapartum-fetal-monitoring-guidelines)<br><br>We recognise that the systematic reviews cited provide insight into the benefits and challenges of applying the principles of track-and-trigger systems. As the reviews highlight, the principles are likely to benefit practice, but only if they are applied to systems that are co-designed with users and implemented using appropriate support to ensure fidelity – as described in the third paragraph of the introduction. This also provides the rationale for our surveys – a first step as part of early and continued engagement of healthcare professionals during development of a new system. We do not understand the reference to an elephant in the room.

	PTTT mandatory across all paediatric units are not supported by the evidence base.<br>Reference 13 states:	
	AUTHORS' CONCLUSIONS: Use of TTS/EWS in maternity care … may result in little to no difference in neonatal death. Both of the included studies were conducted in low-resource settings where the use of TTS/EWS might potentially confer a different effect to TTS/EWS use in high-resource settings. Further highquality trials in high- and middle-resource settings, as well as in discrete populations of high- and low-risk women, are required. The litte evidence there is ("little to no difference in neonatal death") is even in the opposite direction: OR 1.06, 95% CI 0.62 to 1.84.<br>Now, if it is not the claim made in the abstract that motivated the research project, what did then motivate the	

	project? Is there some sort of elephant in the room? I will address this issue in the document Comments solely to the author(s).	
	**Reviewer 3**	
R3-1	Page 6, lines 53 - 56, mentions PPI. However, the citation here is misleading; this study did not involve service users.	Thank you for your review of the paper. The citation – our paper describing the co-design of a clinical practice tool informed by the findings of the surveys presented in the current paper – includes extensive description of how maternity service users were involved in the co-design processes. Please see "Step 1" and "Step 5" in the results section of this paper (https://qualitysafety.bmj.com/content/33/4/258).
R3-2	Research into the consistency of approach to the deterioration of a baby's condition during labour is laudable and much needed. However, the language in this draft paper tends to disembody the pregnant woman or birthing person. I would suggest that within the introduction, the authors signal the importance of autonomy and agency and the need for clinicians to meaningfully discuss their concerns about a deteriorating picture during labour with those in labour and their people.	Thank you for highlighting the importance of research into this area. We have further highlighted the importance of addressing concerns to listen better to families and involve them in their own care in the revised introduction (2nd paragraph) and discussion (page 14).

VERSION 2 - REVIEW

Reviewer 1

Name Evans, Mark I.

Affiliation Icahn School of Medicine at Mount Sinai

Date **23-Dec-2024**

COI

This version attempts to address some of the limitations of the original submission and does make it somewhat more reasonable. Ironically, they repeatedly discuss the concept of formally including other factors into the interpretation but do not even reference the one system (Fetal Reserve Index) about which there are over a dozen publications in its early development that lay out the foundation for such an approach.

VERSION 2 - AUTHOR RESPONSE

	**Reviewer 1**	
R1-1	This version attempts to address some of the limitations of the original submission and does make it somewhat more reasonable. Ironically, they repeatedly discuss the concept of formally including other factors into the interpretation but do not even reference the one system (Fetal Reserve Index) about which there are over a dozen publications in its early development that lay out the foundation for such an approach.<br><br>If you have selected 'Yes' above, please provide details of any competing interests.: I have patents on the Fetal Reserve Index which is mentioned in my review	Thank you for your further review of the paper.<br><br>We have referenced to the Fetal Reserve Index in the discussion of the paper as an example of a tool/approach to improve the integration of fetal heart rate features and other intrapartum risk factors. This includes reference to the following two papers:<br><br>60. Evans MI, Britt DW, Evans SM, Devoe LD. Improving the interpretation of electronic fetal monitoring: the fetal reserve index. Am J Obstet Gynecol. 2023;228:S1129-s43.<br>61. Devoe LD, Britt DW, Evans MI. Reframing antepartum and intrapartum surveillance. Best Pract Res Clin Obstet Gynaecol. 2024;97:102538.
